# Utility of Tumor Suppressor E2F Target Gene Promoter Elements to Drive Gene Expression Specifically in Cancer Cells

**DOI:** 10.3390/cells14241953

**Published:** 2025-12-09

**Authors:** Kenta Kurayoshi, Masakazu Tanaka, Rinka Nakajima, Yaxuan Zhou, Mashiro Shirasawa, Mariana Fikriyanti, Jun-ichi Fujisawa, Ritsuko Iwanaga, Andrew P. Bradford, Keigo Araki, Kiyoshi Ohtani

**Affiliations:** 1 Department of Biomedical Sciences, School of Biological and Environmental Sciences, Kwansei Gakuin University, 1 Gakuen Uegahara, Sanda 669-1330, Hyogo, Japan; kuraken0901@gmail.com (K.K.); hnj51097@kwansei.ac.jp (R.N.); gtk53096@kwansei.ac.jp (Y.Z.); icf08267@kwansei.ac.jp (M.S.); hsj19688@kwansei.ac.jp (M.F.); 2 Division of Neuroimmunology, Joint Research Center for Human Retrovirus Infection, Kagoshima University, Kagoshima 890-8544, Kagoshima, Japan; tanakam@m.kufm.kagoshima-u.ac.jp; 3 Department of Microbiology, Kansai Medical University, Hirakata 570-8506, Osaka, Japan; fujisawa@hirakata.kmu.ac.jp; 4 Department of Obstetrics and Gynecology, University of Colorado School of Medicine, Anschutz Medical Campus, 12700 East 19th Avenue, Aurora, CO 80045, USA; ritsuko.iwanaga@cuanschutz.edu (R.I.); andy.bradford@cuanschutz.edu (A.P.B.); 5 Department of Morphological Biology, Ohu University School of Dentistry, 31-1 Misumido Tomitamachi, Koriyama 963-8611, Fukushima, Japan; k-araki@den.ohu-u.ac.jp

**Keywords:** deregulated E2F, pRB, ARF, TAp73, cancer cell-specific, normal proliferating cells

## Abstract

The transcription factor E2F is the principal target of the tumor suppressor pRB. In almost all cancers, pRB function is disabled due to oncogenic changes, leading to enhanced E2F activity, thereby facilitating aberrant cell proliferation. Enhanced E2F activity has been utilized to drive gene expression preferentially in cancer cells using E2F target promoters, such as the E2F1 promoter. However, these promoters are also activated by physiological E2F activity in normal proliferating cells, resulting in gene expression in normal proliferating cells. In contrast, promoters of tumor suppressor genes, such *ARF* and *TAp73*, are activated by deregulated E2F activity, induced by loss of pRB control, but not by physiological E2F activity, induced by growth stimulation, thereby providing a mechanism to drive expression specifically in cancer cells. Here we show artificial promoters, in which E2F-responsive elements of the *TAp73* gene are tandemly connected to the ARF core promoter, exhibited higher cancer cell specificity than E2F1, hTERT, or ARF promoters. Moreover, adenoviruses driving a cytotoxic gene using these artificial promoters showed cancer cell-specific cytotoxicity and inhibited tumor growth in a xenograft mouse model. These results indicate utility of tumor suppressor gene promoter elements to drive gene expression specifically in cancer cells.

## 1. Introduction

The major obstacles limiting radical treatment of cancer are side effects caused by current therapies, such as radiation and chemotherapy. These therapies kill cancer cells mainly by damaging DNA, inhibiting metabolism or mitosis. Thus, they preferentially damage growing cells, such as cancer cells, which aberrantly proliferate. However, they also impact normally growing cells, such as epithelial and hematopoietic cells. To avoid the consequent side effects, we need to specifically target cancer cells while preserving normally growing cells. For this purpose, several new approaches have been undertaken. One promising strategy is to utilize a cancer cell-specific promoter to express a cytotoxic gene (suicide gene therapy) or a viral gene required for replication of an oncolytic virus (oncolytic virotherapy). By driving expression of a suicide gene, such as *HSV-TK*, or a pro-apoptotic gene, such as *Bax*, under the control of cancer cell-specific promoters, the gene is selectively expressed in cancer cells to induce cell death [1,2,3,4]. Similarly, by driving expression of a viral gene that is essential for replication of an oncolytic virus, under the control of cancer cell-specific promoters, viral replication and consequent cell lysis are restricted to cancer cells [5,6,7,8,9,10,11,12,13]. In these approaches, the efficacy of treatment and the occurrence of side effects depend on the relative activity of the promoter in cancer cells and in normally growing cells, respectively. Therefore, a promoter with high cancer cell specificity should be used for optimal therapeutic efficacy. Ideally the promoter should exhibit high activity in a variety of cancer cell types for wide therapeutic utility of the promoter, but low activity in normally growing cells, to avoid side effects. The hTERT and E2F1 promoters have been frequently used for this purpose [3,4,5,14,15,16,17,18,19].

hTERT is a catalytic subunit of telomerase for reverse transcription, which is required for immortalization of cells. Expression of hTERT is primarily limited to stem cells, and it is not expressed in most somatic cells. However, hTERT expression is induced in various cancers, concomitant with immortalization of cancer cells [20]. Consequently, the hTERT promoter exhibits high activity in cancer cells. However, considering that hTERT is expressed in stem cells and in some somatic cells, such as activated T-cells [21], the hTERT promoter may also be highly active in these normal cells.

E2F1 is a family member of the transcription factor E2F, which plays key roles in cell proliferation by activating a group of growth-related genes. E2F activity is mainly regulated by binding of the RB family proteins (pRB, p107, and p130; referred to as RB) [22,23,24,25,26]. The ability of RB to bind to and inhibit E2F is regulated by phosphorylation, mediated by cyclin-dependent kinases (CDKs). Hypo-phosphorylated RB binds to and inhibits E2F, whereas hyper-phosphorylated RB loses these abilities. Accordingly, RB mediated inhibition of E2F is regulated by upstream signaling pathway such as growth signals, leading to induction of cyclins and activation of CDK, and growth inhibitory signal transduction pathways that lead to induction of CDK inhibitors and suppression of CDK (the RB pathway). Among the RB family members, pRB plays central roles in tumor suppression [27,28]. Hence in almost all cancers, the RB pathway is disabled by oncogenic changes. These include deletion or mutation of pRB or the CDK inhibitor p16^INK4a^, activating mutations of growth signal transducers such as Ras, over-expression of cyclin D1, or mutation of CDK4 rendering it insensitive to CDK inhibitors [22,25,26,29,30]. Consequently, pRB is functionally inactivated and transcriptional activity of E2F is enhanced (deregulated E2F activity). Expression of the *E2F1* gene, one of eight E2F family genes, is induced at the G1/S boundary of the cell cycle, by E2F itself [31], resulting in high E2F1 promoter activity in a wide variety of cancers. Hence the E2F1 promoter has been utilized in oncolytic virotherapy such as oncolytic adenovirus CG0070 [5,14,15,16,17,18,19,32,33,34,35,36,37]. However, the E2F1 promoter is also activated by physiological E2F activity, induced by growth stimulation, and consequently is also highly active in normal proliferating cells [31]. This paradigm also applies for promoters of other growth-related E2F target genes. Therefore, any approach using the E2F1 promoter or other growth-related E2F target gene promoters may also affect normal proliferating cells. To avoid such side effects, the ideal therapeutic targeting promoter should have high activity in cancer cells but not in normal proliferating cells.

Growth-related E2F target genes such as *E2F1* have typical E2F binding sequences (TTT^C^/_G_^G^/_C_CGC) and are activated not only by deregulated E2F activity but also by physiological E2F activity induced by growth stimulation. In contrast, the tumor suppressor E2F target genes, such as *ARF* and *TAp73*, which have atypical E2F response elements (GC repeats), are specifically activated by deregulated E2F activity induced by forced inactivation of RB, which mimics dysfunction of the RB pathway, but not by growth stimulation-induced physiological E2F activity [38,39]. E2F requires heterodimeric partner DP for activation of growth-related genes. This is because E2F strictly depends on DP to bind with high affinity to typical E2F binding sequences of growth-related genes. In contrast, E2F1 binding and activation of atypical E2F response element of the *ARF* gene does not depend on DP [40]. Hence, although the exact molecular nature of deregulated E2F has yet to be elucidated, deregulated E2F activity that activates the *ARF* and *TAp73* genes is thought to be distinct from simply enhanced E2F activity. ARF inactivates HDM2, an upstream negative regulator of p53, leading to activation of p53, thereby promoting cell cycle arrest or apoptosis to protect cells from tumorigenesis [41]. TAp73 is a member of the p53 family that can activate p53 target genes and induce apoptosis in a p53-independent manner [42]. The *TAp73* gene is a direct target of E2F [39,43,44,45]. The *ARF* and *TAp73* genes are selectively activated by deregulated E2F activity but not by physiological E2F activity induced by growth stimulation [38,39]. Thus, normal proliferating cells lack the deregulated E2F activity required to activate the ARF or TAp73 promoter. In contrast, in almost all cancers, the p53 pathway is also disabled [30], allowing cancer cells to survive in the presence of deregulated E2F activity. Hence, deregulated E2F activity, which activates the *ARF* and *TAp73* genes, is expected to exist specifically in cancer cells [38,39]. These observations suggest that the ARF and TAp73 promoters show low activity in normally growing cells and high activity in cancer cells, thereby conferring an advantage over promoters of growth-related E2F target genes, such as the E2F1 promoter, in driving cancer cell-specific gene expression [46]. Indeed, the ARF promoter exhibited higher cancer cell specificity than the E2F1 promoter in driving a suicide gene expression [47]. However, whether this is due to deregulated E2F activity in cancer cells or is simply a unique property of the ARF promoter has yet to be elucidated, since the *ARF* gene functions as a sensor of a variety of oncogenic changes [48,49]. To examine the feasibility of using deregulated E2F activity and tumor suppressor gene promoters to drive gene expression specifically in cancer cells, we constructed artificial promoters, in which E2F response elements of the TAp73 promoter (ERE73s) are tandemly conjugated to the ARF core promoter, and compared their cancer cell specificity with those of promoters harboring E2F binding site mutations in ERE73s. The artificial promoters showed significantly higher cancer cell specificity compared to E2F1, hTERT, and ARF promoters, indicating that, by artificially modifying tumor suppressor gene promoters, we can enhance cancer cell-specific gene expression. In addition, introduction of point mutations into the E2F binding sites of ERE73s abolished the cancer cell-specific activity, indicating that enhanced specificity is due to deregulated E2F activity in cancer cells. Moreover, recombinant adenoviruses expressing *HSV-TK*, a suicide gene, under the control of the ERE73s-ARF artificial promoters showed cancer cell-specific killing in vitro and suppressed tumor growth in vivo in a xenograft mouse model. These results indicate utility of tumor suppressor gene promoter elements to drive cytotoxic gene expression, specifically in cancer cells.

## 2. Materials and Methods

### 2.1. Cell Culture

Human normal fibroblasts (human foreskin fibroblasts: HFFs (RRID: CVCL_XB54), obtained from ATCC) and human cancer cell lines (human osteosarcoma cell line Saos-2 (RRID: CVCL_0548), human hepatocellular carcinoma cell line HLF (RRID: CVCL_2947)) were cultured in Dulbecco’s modified Eagle medium (DMEM) (FUJIFILM Wako, Osaka, Japan) containing 10% fetal calf serum (FCS). Other human cancer cell lines (human bladder carcinoma cell line 5637 (RRID: CVCL_0126), human colon adenocarcinoma cell line DLD-1 (RRID: CVCL_0248)) were cultured in RPMI 1640 medium (FUJIFILM Wako) containing 10% FCS.

### 2.2. Plasmid

pARF-Luc (−736), pARF (−13)-Luc, and pE2F1-Luc (−728) have been described [31,38,39]. pARF-Luc (−736) contains the −736 to +49 region of ARF promoter in pGL3-Basic (Promega, Madison, WI, USA). pARF (−13)-Luc is a 5′ deletion mutant of pARF-Luc (−736) lacking regulatory region upstream of −13. pE2F1-Luc (−728) contains the −728 to +28 region of E2F1 promoter in pGL3-Basic (Promega). phTERT-Luc (−378) was made by cloning the −378 to +58 region of hTERT promoter into pGL3-Basic (Promega). pERE73 (1 + 2)-ARF (−13)-Luc and pERE73 (3 + 4)-ARF (−13)-Luc were made by cloning 3 copies of ERE73 (1 + 2) or 5 copies of ERE73 (3 + 4) from TAp73 promoter [39] into pARF (−13)-Luc [39]. pE2WTx4-ARF (−13)-Luc and pE2MTx4-ARF (−13)-Luc were generated by subcloning E2WTx4 and E2MTx4 sequences of pE2WTx4-Luc and pE2MTx4-Luc [50] into pARF (−13)-Luc [39], respectively. The expression vectors for E2F1, E2F2, E2F3a (pENTR-E2F1, pENTR-E2F2, pENTR-E2F3a), the constitutively active form of pRB (pPSM.7-LP (Neo-Bam)), and the Δ2-11 form of adenovirus 12S E1a (pENTR-12SE1a (Δ2-11)) have been described [39]. pRL-CMV is a CMV promoter-driven Renilla luciferase vector (Promega). pENTR-CMV-HSV-TK was generated by subcloning the *HSV-TK* gene from pTK5 (RIKEN BRC, Tsukuba, Japan) into pENTR-CMV [39]. pENTR-ERE73 (1 + 2)-ARF (−13)-TK and pENTR-ERE73 (3 + 4)-ARF (−13)-TK were generated by replacing the CMV promoter of pENTR-CMV-HSV-TK with ERE73 (1 + 2)-ARF (−13) and ERE73 (3 + 4)-ARF (−13) from pERE73 (1 + 2)-ARF (−13)-Luc and pERE73 (3 + 4)-ARF (−13)-Luc, respectively. pENTR-pless-TK was generated by deletion of CMV promoter from pENTR-CMV-HSV-TK. pENTR-iRFP720 was generated by subcloning iRFP720 cDNA from pNLS-iRFP720 (addgene #45467) into pENTR-CMV.

### 2.3. Luciferase Assay

Cells were transfected with reporter and effector plasmids using FuGENE 6 (Promega) or PEI Max (Polysciences, Warrington, PA, USA) with the proportion of DNA versus FuGENE 6 or PEI Max 1:3. To control for transfection efficiency, a CMV promoter-driven Renilla luciferase vector pRL-CMV (Promega) was included as an internal control. Luciferase and Renilla luciferase activities were measured using Dual-Luciferase Reporter Assay System (Promega), and luciferase activity was normalized to that of Renilla luciferase. All assays were repeated at least three times and values are shown as means ± SD.

### 2.4. Quantitative Reverse Transcription (qRT)-PCR Analysis

Total RNA was extracted using Isogen (Nippon Gene, Tokyo, Japan) and genomic DNA was digested using Recombinant DNase I (TaKaRa, Shiga, Japan). The first strand cDNA was synthesized using the PrimeScript RT reagent Kit for RT-PCR [AMV] (TaKaRa) using the oligo (dT) primer. Quantitative PCR was performed using KAPA SYBR qPCR Mix (KAPA Biosystems, Wilmington, MA, USA) and Thermal Cycler Dice Real Time System Single (TaKaRa). The expression levels of *HSV-TK* mRNA were adjusted by that of *GAPDH* as an internal control. The following primer sets were used:

*HSV-TK*:
Fw: 5′-AGCAAGA AGCCACGGAAGTC-3′;
Rv: 5′-GGCGGTCGAAGATGAGGGTGA-3′.
*GAPDH*:
Fw: 5′-GGAGTCCACTGGCGTCTTCA-3′;
Rv: 5′-GAGGGGCCATCCACAGTCTT-3′.
*iRFP720*:
Fw: 5′-GGAGGCGGCACAGCTACGAGAACG-3′;
Rv: 5′-GCGGCGAGGGCGAGCAG CAGTC-3′.

### 2.5. Immunoblot Analysis

Immunoblot analysis was carried out as described previously [51]. The antibodies used were anti-E2F3 (sc-878, Santa Cruz Biotechnology, Dallas, TX, USA, 1:300), anti-CDC6 (sc-9964, Santa Cruz, 1:500), anti-Cyclin A (sc-751, Santa Cruz, 1:500), anti-Adenovirus 5 E1a (M58; BD Biosciences Pharmingen, Franklin Lakes, NJ, USA, 1:1000), and anti-β-actin (A1978, SIGMA-Aldrich, St. Louis, MO, USA, 1:2000). The secondary antibodies used were anti-mouse IgG-HRP (Jackson, Scottsdale, CA, USA, 1:1000) and anti-rabbit IgG-HRP (1: 5000, NA934, GE Healthcare, Chicago, IL, USA). The signals were detected using LAS4000 (GE Healthcare) after treatment with ImmunoStar LD (Fujifilm, Tokyo, Japan).

### 2.6. Recombinant Adenovirus

Ad-Con and Ad-12SE1a (Δ2-11) have been described [52]. Ad-pless-TK, Ad-ERE73 (1 + 2)-ARF (−13)-TK, Ad-ERE73 (3 + 4)-ARF (−13)-TK, and Ad-CMV-iRFP720 were generated from pENTR-pless-TK, pENTR-ERE73 (1 + 2)-ARF (−13)-TK, pENTR-ERE73 (3 + 4)-ARF (−13)-TK, and pENTR-iRFP720, respectively, using Vira Power Adenoviral expression system (Thermo Fisher Scientific, Waltham, MA, USA) according to the supplier’s protocol. Infection with recombinant adenoviruses proceeded as previously described [53].

### 2.7. FACS Analysis

Cells were fixed with 70% ethanol and stained with propidium iodide (50 μg/mL) containing RNase (50 μg/mL). Cell samples were analyzed with a FACSCalibur (Becton Dickinson, Franklin Lakes, NJ, USA). All assays were repeated at least three times and values are shown as means ± SD.

### 2.8. Xenograft Assay

The cultured DLD-1 cells were detached and suspended in 0.05 mL RPMI-1640 medium and combined with 0.05 mL Matrigel (BD). The cells (5 × 10^6^ cells in 0.1 mL) were injected subcutaneously into the right flank of athymic 6-week BALB/c nu/nu female mice (CAnN.Cg-Foxn1nu/CrlCrlj). When average tumor volume reached about 400 mm^3^, mice were randomly assigned into 4 groups: PBS, Ad-pless-TK, Ad-ERE73 (1 + 2)-ARF (−13)-TK, or Ad-ERE73 (3 + 4)-ARF (−13)-TK (1 × 10^10^ PFU in 0.1 mL PBS)-injected groups (3 mice per group). From a day after infection, Ganciclovir (50 mg/kg/day) was administered intraperitoneally once per day for 10 days. The tumor sizes were monitored for the following 10 days by tumor volumes. The tumor volumes (mm^3^) were estimated as length × width^2^/2. Tumor weights were measured at 11 days after infection. Animal experiments were approved by the Animal Care and Use Committee of Kansai Medical University (Approval number: 13-110) and Kwansei Gakuin University (Approval number: 2013-24).

### 2.9. Analysis of Liver Function

Eleven days after infection of mice with the recombinant adenoviruses, blood samples were collected from the mice, and aspartate aminotransferase (AST) and alanine transaminase (ALT) levels in plasma were examined (LSI Medience Corporation, Tokyo, Japan).

### 2.10. Histological Analysis

Eleven days after infection of mice with the recombinant adenoviruses, the liver and spleen were collected and fixed with 10% formaldehyde for Hematoxylin Eosin (HE) staining.

### 2.11. Statistical Analysis

Luciferase assays, FACS analyses of dead cells, and qRT-PCR experiments were conducted in biological triplicate. A mouse xenograft model was also implemented in biological triplicate (three mice/group). Data are presented as means ± SD. Statistical comparisons were made using Student’s *t*-test and Bonferroni correction for multiple comparisons. A *p* value < 0.05 was considered as significant.

## 3. Results

### 3.1. ERE73 (1 + 2)-ARF (−13) and ERE73 (3 + 4)-ARF (−13) Promoters Are More Cancer Cell-Specific than hTERT, E2F1, E2WT-ARF (−13), and ARF Promoters

**Figure 1:**
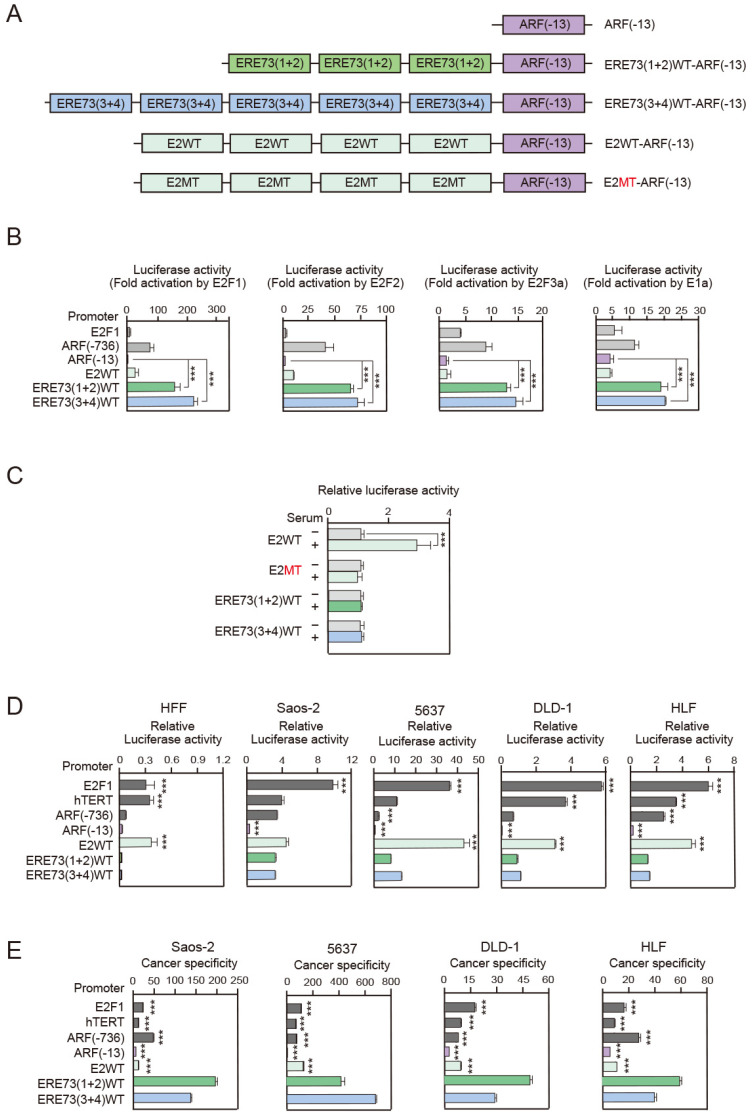
ERE73 (1 + 2)-ARF (−13) and ERE73 (3 + 4)-ARF (−13) exhibit higher cancer cell specificity. (**A**) Schematic representation of ERE73 (1 + 2) or ERE73 (3 + 4) connected to the ARF core promoter pARF (−13). Enhancer region of adenovirus E2 gene (E2), which contains two typical E2F binding sequences, is used as a positive control for response to growth stimulation. (**B**) HFFs were transfected with E2F1 (−728), ARF (−736), pARF (−13), E2WT-ARF (−13), pERE73 (1 + 2)-ARF (−13), or pERE73 (3 + 4)-ARF (−13) reporter plasmid along with expression vector for E2F1, E2F2, E2F3a, or adenovirus E1a. pRL-CMV was included as an internal control. The next day, the cells were washed with PBS, cultured in DMEM containing 0.1% FCS for 1 day, and harvested. Luciferase activity was measured, adjusted by that of Renilla luciferase and presented as fold activations by E2F1, E2F2, E2F3a, or adenovirus E1a. *** *p* < 0.01. (**C**) HFFs were cultured in DMEM containing 0.1% FCS for 1 day and transfected with E2WT-ARF (−13), E2MT-ARF (−13), pERE73 (1 + 2)-ARF (−13), or pERE73 (3 + 4)-ARF (−13) reporter plasmid along with pRL-CMV as an internal control. The cells were cultured in DMEM containing 0.1% FCS for 1 day after transfection, restimulated with serum, and harvested after 20 h. Luciferase activity was measured and adjusted by that of Renilla luciferase and presented as fold activations by serum. *** *p* < 0.01. (**D**) HFFs and cancer cell lines (Saos-2, 5637, DLD-1, and HLF) were transfected with E2F1 (−728), hTERT (−378), ARF (−736), ARF (−13), E2WT, ERE73 (1 + 2)-ARF (−13), or ERE73 (3 + 4)-ARF (−13) reporter plasmid with pRL-CMV as an internal control. The cells were cultured for 1 day after transfection and were harvested. Luciferase was measured, adjusted by that of Renilla luciferase, and presented as relative activity. *** *p* < 0.01 compared to ERE73 (1 + 2)WT or ERE73 (3 + 4)WT. (**E**) Cancer cell specificity was calculated between 4 cancer cell lines (Saos-2, 5637, DLD-1, and HLF) and HFFs as normal cells and is presented as fold activities in cancer cell lines compared to that in HFFs. *** *p* < 0.01 compared to ERE73 (1 + 2) WT or ERE73 (3 + 4) WT.

To explore the potential utility of the promoter elements of the tumor suppressor E2F target genes to drive gene expression specifically in cancer cells, we investigated whether cancer cell specificity can be increased, by constructing artificial promoters combining E2F-responsive elements and core promoters of deregulated E2F target tumor suppressor genes. To this end, we fused three or five tandem repeats of ERE73 (1 + 2) or ERE73 (3 + 4), the E2F-responsive elements of the TAp73 promoter (ERE73s) [39], to the ARF core promoter (ARF (−13)) [38,39], generating ERE73 (1 + 2)WT-ARF (−13) and ERE73 (3 + 4)WT-ARF (−13), respectively (Figure 1A). ARF (−736) contains −736 to +49 region of the ARF promoter and ARF (−13) is a 5′ deletion mutant of ARF (−736) lacking the regulatory region upstream of −13 including E2F-responsive element of ARF promoter (EREA) [38]. Thus ARF (−13) is a core promoter lacking E2F responsiveness [39]. This strategy is based on our observation that the ARF promoter, ARF (−736), showed low basal promoter activity in normally growing cells, and deletion of upstream regulatory region, ARF (−13), further reduced the basal activity. We hypothesized that addition of enhancer elements to core promoters, in a tandemly repeated manner, enhanced responsiveness, such that the artificial promoters exhibit minimal activity in normally growing cells but high activity in cancer cells. We have previously identified 4 E2F-responsive elements in the TAp73 promoter (ERE73-1 through ERE73-4); ERE73 (1 + 2) is a region containing ERE73-1 and -2, and ERE73 (3 + 4) is a region containing ERE73-3 and -4, respectively [39].

First, we examined the response of these artificial promoters to ectopic expression of activator E2Fs (E2F1, E2F2, E2F3a) along with the native ARF promoter, E2F1 promoter, and E2WT-ARF (−13) construct. The combination of ERE73 (1 + 2) or ERE73 (3 + 4) in tandem repeats, conferred significantly enhanced responsiveness to over-expression of activator E2Fs, especially E2F1, compared to the ARF (−13) core promoter construct (Figure 1B). Remarkably, the ERE73 (1 + 2)-ARF (−13) and ERE73 (3 + 4)-ARF (−13) constructs showed higher responsiveness to ectopic expression of E2F1, E2F2, or E2F3a compared to the native ARF promoter, the commonly used E2F1 promoter, and E2WT-ARF (−13) construct, which contains eight copies of typical E2F binding sequences (Figure 1B). This result suggests that the atypical E2F response elements in the tumor suppressor *TAp73* gene are more sensitive to deregulated E2F activity than canonical E2F binding sites. We also examined the response of the ERE73-ARF (−13) constructs to endogenous deregulated E2F activity, induced by forced inactivation of pRB by adenovirus E1a, which mimics dysfunction of the RB pathway, a prototype of oncogenic changes. The combination of ERE73 (1 + 2) or ERE73 (3 + 4) also conferred a significant increase in response to E1a, which was much higher than the ARF, E2F1, and E2WT-ARF (−13) promoters (Figure 1B). These results suggest that combination of E2F-responsive elements with a core promoter of tumor suppressor genes can generate an artificial promoter with higher sensitivity to deregulated E2F activity than the commonly used E2F1 promoter (Figure 1B).

We next examined the effects of physiological E2F activity, induced by serum, which stimulates growth of human normal fibroblasts, on these promoter constructs. E2WT-ARF (−13), which has four tandem repeats of the enhancer region of the adenovirus E2 gene containing two typical E2F binding sites, and E2MT-ARF (−13), which has point mutations in these canonical E2F binding sites, were used as positive and negative controls for growth stimulation, respectively (Figure 1A). While E2WT-ARF (−13) exhibited significant activation by serum stimulation, ERE73 (1 + 2)-ARF (−13) and ERE73 (3 + 4)-ARF (−13) did not show a serum response and were comparable to the mutant E2MT-ARF (−13) (Figure 1C). This indicates that the ERE73 (1 + 2)-ARF (−13) and ERE73 (3 + 4)-ARF (−13) constructs are activated by deregulated E2F activity, but not by physiological E2F activity induced by serum stimulation, suggesting that these artificial promoters are minimally active in normally growing cells but exhibit high activity in cancer cells.

The increased sensitivity of the ERE73 (1 + 2)-ARF (−13) and ERE73 (3 + 4)-ARF (−13) promoter constructs to deregulated E2F activity, together with their unresponsiveness to physiological E2F activity induced by growth stimulation, suggested that they exhibit high promoter activity in cancer cells and low activity in normally growing cells, resulting in enhanced cancer cell specificity. To test this possibility, we examined the activity of the ERE73 (1 + 2)-ARF (−13) and ERE73 (3 + 4)-ARF (−13) constructs, along with that of the native ARF, E2F1, and hTERT promoters, and the E2WT-ARF (−13) construct. As a representative normally growing cell, we used human normal fibroblasts (HFFs). The human osteosarcoma cell line Saos-2 served as a representative cancer cell line. Saos-2 cells lack p53 and contain a nonfunctional form of pRB (p95) with a C-terminal truncation [54]. In normal human fibroblasts (HFFs), the activity of the ARF promoter was much lower than that of the hTERT and E2F1 promoters. Core ARF (−13) promoter activity was even lower, approximately one-third that of the native ARF promoter. Notably, addition of ERE73 (1 + 2) and ERE73 (3 + 4) did not increase basal promoter activity in HFFs (Figure 1E). Consequently, ERE73 (1 + 2)-ARF (−13) and ERE73 (3 + 4)-ARF (−13) also showed far lower activity than the E2F1 and hTERT promoters (Figure 1D). This is likely due to normally growing cells lacking deregulated E2F activity required to activate ERE73s. In contrast, addition of adenovirus E2 enhancer containing typical E2F binding sequences, E2WT-ARF (−13) greatly enhanced promoter activity to levels equivalent to those of the E2F1 and hTERT promoters in HFFs. This is consistent with physiological E2F activity that targets typical E2F binding sequences in growth-related genes, which may underlie the high activity of the E2F1 promoter in normally growing cells. In the cancer cell line Saos-2, ARF (−13) still showed very low activity, which was greatly enhanced by addition of ERE73 (1 + 2) and ERE73 (3 + 4) (Figure 1D). Consequently, activity of ERE73 (1 + 2)-ARF (−13) and ERE73 (3 + 4)-ARF (−13) was comparable to that of the hTERT and ARF promoters and E2WT-ARF (−13), although lower than that of the E2F1 promoter (Figure 1D). These results suggest that ERE73 (1 + 2)-ARF (−13) and ERE73 (3 + 4)-ARF (−13) have similar activity to the hTERT, ARF, and E2WT-ARF (−13) promoters in Saos-2 cancer cells, while exhibiting much weaker activity in normally growing cells. Thus, we predicted that cancer cell specificity of the ERE73s-ARF (−13) constructs would be correspondingly increased. To examine this possibility and determine the relative cancer cell-specific activity of each promoter construct, we defined cancer cell specificity as follows:

Cancer cell specificity = activity in a cancer cell line/activity in HFFs

In Saos-2 cells, the ERE73 (1 + 2)-ARF (−13) and ERE73 (3 + 4)-ARF (−13) promoters exhibited higher cancer cell specificity than that of the hTERT, ARF, or E2WT-ARF (−13) promoters, and even exceeded that of the E2F1 promoter (Figure 1E). The higher cancer cell specificity of the ERE73s-ARF (−13) is primarily a reflection of the relatively low activity of the ERE73s-ARF constructs in normal HFFs, compared to the hTERT, ARF, or E2WT-ARF (−13) promoters (Figure 1D). This would be therapeutically beneficial for preserving normally growing cells.

To extend these observations, we examined promoter activity and cancer cell specificity of these promoter constructs in additional cancer cell lines originating from different tissue types and with different status of the RB and p53 pathways, 5637, DLD-1, and HLF. 5637 is a urinary bladder carcinoma cell line, which has mutations in both pRB and p53. DLD1 is a colon adenocarcinoma cell line with mutations in adenomatous coli (APC), K-Ras, and p53. HLF is a hepatocellular carcinoma cell line, which expresses mutant p53, but mutations in the RB pathway have not been reported. Similarly to the results in Saos-2 cells, although the ERE73 (1 + 2)-ARF (−13) and ERE73 (3 + 4)-ARF (−13) promoters showed comparable to or lower activity than E2F1, hTERT, ARF, and E2WT-ARF (−13) constructs (Figure 1E), they exhibited higher cancer cell specificity than E2F1, hTERT, and ARF promoters and the E2WT-ARF (−13) construct across all three cancer cell lines (Figure 1E). These results demonstrate that the ERE73 (1 + 2) and ERE73 (3 + 4) elements confer superior cancer cell specificity on the ARF (−13) core promoter, compared to the E2F1, hTERT, and native ARF promoters and E2WT-ARF (−13) construct, in these cancer cell lines.

### 3.2. Deregulated E2F Activity Contributes to High Cancer Specificity of ERE73 (1 + 2)-ARF (−13) and ERE73 (3 + 4)-ARF (−13)

**Figure 2:**
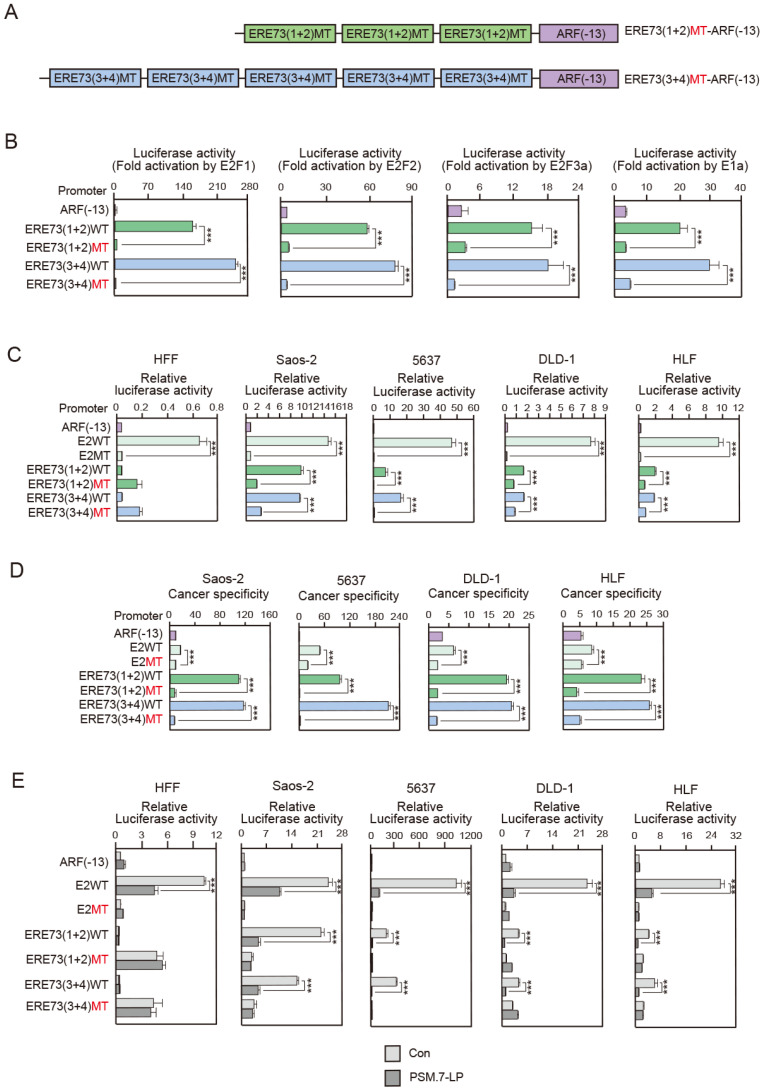
Deregulated E2F activity in cancer cells contributes to cancer cell-specific activity of the ERE73 (1 + 2)-ARF (−13) and ERE73 (3 + 4)-ARF (−13) promoters. (**A**) Schematic representation of E2F site mutants of E2, ERE73 (1 + 2) and ERE73 (3 + 4) connected to ARF core promoter ARF (−13). (**B**) HFFs were transfected with ARF (−13), pERE73 (1 + 2)-ARF (−13), pERE73 (1 + 2) MT-ARF (−13), pERE73 (3 + 4)-ARF (−13), and pERE73 (3 + 4) MT-ARF (−13) along with expression vector for E2F1, E2F2, E2F3a, or adenovirus E1a. pRL-CMV was included as an internal control. The cells were cultured for 1 day after transfection and were harvested. Luciferase activity was measured, adjusted by that of Renilla luciferase, and presented as fold activations by E2F1, E2F2, E2F3a, or adenovirus E1a. *** *p* < 0.01. (**C**) HFFs and cancer cells (Saos-2, 5637, DLD-1 and HLF) were transfected with ARF (−13), E2WT-ARF (−13), E2mt-ARF (−13), ERE73 (1 + 2)-ARF (−13), ERE73 (1 + 2) MT-ARF (−13), ERE73 (3 + 4)-ARF (−13), or ERE73 (3 + 4) MT-ARF (−13) reporter. pRL-CMV was included as an internal control. The cells were cultured for 1 day after transfection and were harvested. Luciferase activity was measured, adjusted by that of Renilla luciferase, and presented as relative luciferase activities. *** *p* < 0.01. (**D**) Cancer cell specificity was calculated between 4 cancer cell lines (Saos-2, 5637, DLD-1, and HLF) and HFFs as normal cells and is presented as fold activities in cancer cell lines compared to that in HFFs. *** *p* < 0.01. (**E**) HFFs and cancer cells (Saos-2, 5637, DLD-1 and HLF) were transfected with ARF (−13), E2WT-ARF (−13), E2MT-ARF (−13), ERE73 (1 + 2)-ARF (−13), ERE73 (1 + 2) MT-ARF (−13), ERE73 (3 + 4)-ARF (−13), or ERE73 (3 + 4) MT-ARF (−13) reporter with the expression vector for the constitutively active form of pRB, PSM.7-LP. pRL-CMV was included as an internal control. The cells were cultured for 1 day after transfection and were harvested. Luciferase activity was measured, adjusted by that of Renilla luciferase, and presented as relative luciferase activities. *** *p* < 0.01.

To determine whether the increased cancer cell specificity of ERE73 (1 + 2)-ARF (−13) and ERE73 (3 + 4)-ARF (−13) is due to deregulated E2F activity in the cancer cell lines, we introduced point mutations (MTs) in the ERE73 (1 + 2) and ERE73 (3 + 4) response elements, which successfully abrogated E2F responsiveness in our previous report (Figure 2A) [39]. We first examined responsiveness of ERE73 (1 + 2) MT-ARF (−13) and ERE73 (3 + 4) MT-ARF (−13) to over-expression of E2F1, E2F2, and E2F3a, and expression of E1a. The mutations essentially abrogated activation by activator E2Fs and E1a, confirming that mutation of the ERE73s successfully abolished E2F responsiveness (Figure 2B). We next compared the promoter activity of ERE73 (1 + 2) MT-ARF (−13) and ERE73 (3 + 4) MT-ARF (−13) with that of the ERE73 wild type in both HFFs and cancer cell lines (Saos-2, 5637, DLD-1, and HLF). Remarkably, ERE73 (1 + 2) MT-ARF (−13) and ERE73 (3 + 4) MT-ARF (−13) mutants displayed lower promoter activity than their analogous wild-type constructs exclusively in the cancer cell lines (Saos-2, 5637, DLD-1, and HLF), while no significant downregulation of promoter activity was observed in human normal fibroblasts (HFFs) (Figure 2C). Consequently, this disparity in promoter activity resulted in a loss of cancer cell specificity of ERE73 (1 + 2) MT-ARF (−13) and ERE73 (3 + 4) MT-ARF (−13) in comparison to their wild-type constructs (Figure 2D). These observations support the notion that deregulated E2F activation via the ERE73s elements significantly contributes to the enhanced cancer cell specificity observed in ERE73 (1 + 2)-ARF (−13) and ERE73 (3 + 4)-ARF (−13) constructs.

To further elucidate the role of deregulated E2F activity in the elevated cancer cell specificity of ERE73 (1 + 2)-ARF (−13) and ERE73 (3 + 4)-ARF (−13), we examined whether repression of E2F activity by PSM.7-LP, a constitutively active form of pRB, could diminish the cancer cell-specific activity of both ERE73s-ARF constructs. We evaluated the effects of PSM.7-LP on the activity of ERE73 (1 + 2)-ARF (−13), ERE73 (3 + 4)-ARF (−13), and E2WT-ARF (−13), and the analogous mutant constructs, in human normal fibroblasts (HFFs) and the cancer cell lines. In HFFs, E2WT-ARF (−13), which has typical E2F binding sites, was repressed by PSM.7-LP, indicating that physiological E2F activity, induced by serum stimulation, was suppressed by constitutively active pRB (Figure 2E). In contrast, ERE73 (1 + 2)-ARF (−13) and ERE73 (3 + 4)-ARF (−13) were unaffected by PSM.7-LP (Figure 2E). This supports the notion that ERE73 (1 + 2)-ARF (−13) and ERE73 (3 + 4)-ARF (−13) are not activated by physiological E2F in HFFs. In contrast, in the cancer cell lines (Saos-2, 5637, DLD-1, and HLF), not only E2WT-ARF (−13) but also ERE73 (1 + 2)-ARF (−13) and ERE73 (3 + 4)-ARF (−13) were repressed by PSM.7-LP (Figure 2E). Notably, activity of the corresponding ERE73 mutant constructs was not impacted by PSM.7-LP in the cancer cell lines (Figure 2E). These observations imply that PSM.7-LP diminished the activity of ERE73 (1 + 2)-ARF (−13) and ERE73 (3 + 4)-ARF (−13) through suppression of deregulated E2F activity in the cancer cell lines, supporting the notion that deregulated E2F activity confers high cancer cell specificity on the ERE73 (1 + 2)-ARF (−13) and ERE73 (3 + 4)-ARF (−13) constructs.

### 3.3. Ad-ERE73 (1 + 2)-ARF (−13)-TK and Ad-ERE73 (3 + 4)-ARF (−13)-TK Virus Vectors Induce Cell Death Specifically in Cancer Cells

**Figure 3:**
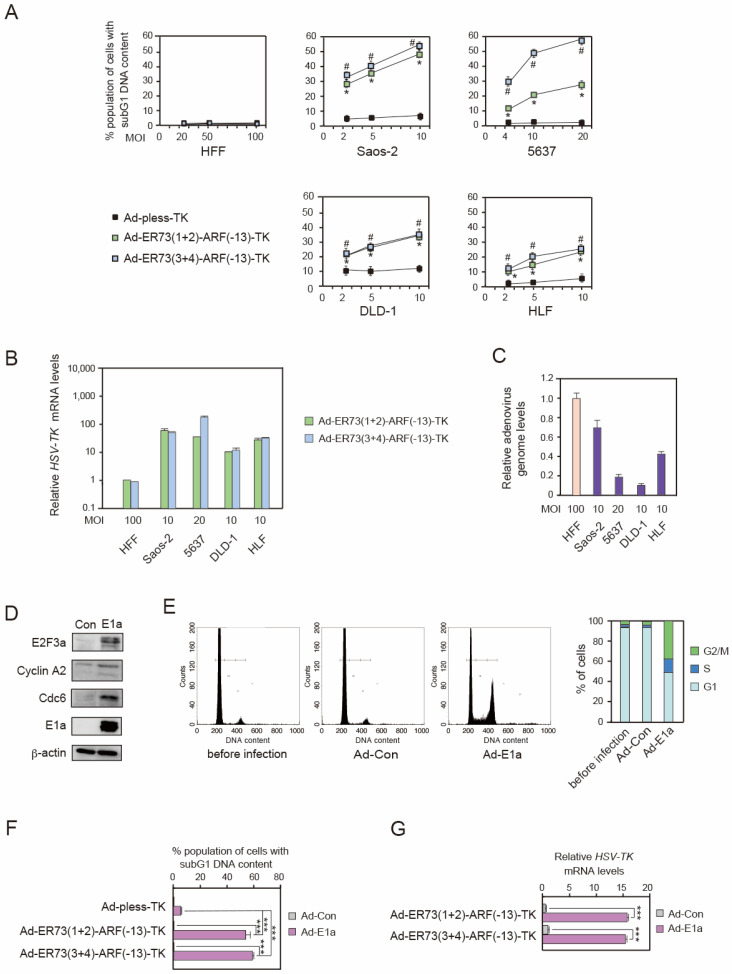
Ad-ERE73 (1 + 2)-ARF (−13)-TK and Ad-ERE73 (3 + 4)-ARF (−13) drive cancer cell-specific gene expression. (**A**) HFFs and cancer cells (Saos-2, 5637, DLD-1, and HLF) were infected with Ad-pless-TK, Ad-ERE73 (1 + 2)-ARF (−13)-TK, and Ad-ERE73 (3 + 4)-ARF (−13) or Ad-CMV-TK with increasing MOI. The cells were cultured for 5 days in DMEM or RPMI in the presence of Ganciclovir (GCV) (50 μM) and harvested. The percentage of cells with subG1 DNA content was determined by FACS analysis after staining DNA with propidium iodide (PI). Percentages of cells with subG1 DNA content are presented for each cell line. * and # *p* < 0.01 compared to Ad-pless-TK. (**B**) Under the same conditions, expression levels of HSV-TK mRNA were examined by qRT-PCR. HSV-TK mRNA levels are normalized to that of GAPDH as an internal control. Relative expression levels are shown with that of ERE73 (1 + 2)-ARF (−13) in HFFs set as 1. (**C**) HFFs and cancer cells (Saos-2, 5637, DLD-1, and HLF) were infected with Ad-CMV-iRFP720. The cells were cultured for 5 days in DMEM or RPMI and harvested. After purification of high-molecular-weight DNA, the levels of adenovirus genome were examined by qPCR using a primer set specific for iRFP720. (**D**) HFFs were cultured in DMEM containing 0.1% FCS for 2 days and infected with Ad-12SE1a (Δ2-11) with MOI 200. The cells were cultured for 24 h after infection and were harvested. Expression of E2F target gene products was examined by Western blot analysis. (**E**) HFFs were cultured in DMEM containing 0.1% FCS for 2 days and infected with Ad-12SE1a (Δ2-11). The cells were cultured in DMEM 0.1% FCS for 2 days after infection and harvested. Cell cycle distribution of the cells was examined by FACS analysis after staining DNA with PI. (**F**) HFFs were cultured in DMEM containing 0.1% FCS for 2 days and infected with Ad-12SE1a (Δ2-11) along with Ad-pless-TK, Ad-ERE73 (1 + 2)-ARF (−13)-TK, or Ad-ERE73 (3 + 4)-ARF (−13)-TK. The cells were further cultured under the serum starved condition for 5 days after infection in the presence of Ganciclovir (50 μM) and harvested. Dead cells were measured by FACS analysis after staining with PI and percentages of cells with subG1 DNA content are presented. *** *p* < 0.01. (**G**) Under the same condition, expression of *HSV-TK* mRNA was examined by qRT-PCR and relative mRNA levels are presented. *** *p* < 0.01.

To explore the potential application of the ERE73s-ARF (−13) constructs in cancer cell-specific gene therapy, we generated recombinant adenovirus expressing *HSV-TK* under the control of the ERE73 (1 + 2)-ARF (−13) and ERE73 (3 + 4)-ARF (−13) promoter constructs, generating Ad-ERE73 (1 + 2)-ARF (−13)-TK and Ad-ERE73 (3 + 4)-ARF (−13)-TK, respectively. We then compared the relative cytotoxicity of these viruses in normally growing cells (HFFs) and cancer cell lines (Saos-2, 5637, DLD-1, and HLF), with varying multiplicity of infection (MOI). Since ERE73 (1 + 2)-ARF (−13) and ERE73 (3 + 4)-ARF (−13) showed extremely low activity in HFFs, HFFs were infected at one-order-higher MOI. Cells were cultured in the presence of Ganciclovir in the media, which is metabolized by HSV-TK into an active form, resulting in cell death. Dead cells were identified and quantitated as those with subG1 DNA content by FACS analysis. In normal HFFs, the population of cells with sub-G1 DNA content in cells infected with Ad-ERE73 (1 + 2)-ARF (−13)-TK and ERE73 (3 + 4)-ARF (−13)-TK was extremely low (less than 3%), even with increasing MOI, and was similar to those infected with the control Ad-pless-TK virus, which lacks *HSV-TK* expression (Figure 3A), indicating that Ad-ERE73 (1 + 2)-ARF (−13)-TK and Ad-ERE73 (3 + 4)-ARF (−13)-TK do not show cytotoxicity in HFFs at this range of MOI. In contrast, in the four cancer cell lines (Saos-2, 5637, DLD-1, and HLF), cells infected with Ad-ERE73 (1 + 2)-ARF (−13)-TK and ERE73 (3 + 4)-ARF (−13)-TK demonstrated a significantly higher proportion of cells with subG1 DNA content compared to Ad-pless-TK with increasing MOI (Figure 3A). To confirm the higher expression of the *HSV-TK* gene in cancer cell lines compared to normal HFFs, we examined *HSV-TK* mRNA levels by qRT-PCR. Consistent with the high cytotoxicity of Ad-ERE73 (1 + 2)-ARF (−13)-TK and Ad-ERE73 (3 + 4)-ARF (−13)-TK in cancer cell lines, the *HSV-TK* gene is expressed in cancer cells at levels 10- to 100-fold greater than that in normal HFFs, even at an order of magnitude lower viral MOI (Figure 3B). To exclude the possibility that low expression of *HSV-TK* in HFFs was due to low infection efficiency in normal cells, we examined the adenovirus genome copy number present in the cells by qPCR. Notably, the copy number of the adenoviral genome in normal cells (HFFs), following infection, was significantly greater than that in the cancer cell lines (Saos-2, 5637, DLD-1, and HLF), consistent with the higher MOI (Figure 3C). These observations strongly suggest that ERE73 (1 + 2)-ARF (−13) and ERE73 (3 + 4)-ARF (−13) promoters can effectively drive cancer cell-specific gene expression.

To extend these observations, we examined whether Ad-ERE73 (1 + 2)-ARF (−13)-TK and Ad-ERE73 (3 + 4)-ARF (−13)-TK could kill normal HFFs transduced with adenovirus E1a, which binds to and inactivates all RB family members, thus mimicking dysfunction of the RB pathway. Adenovirus-mediated expression of the Δ2-11 form of 12SE1a in serum starved HFF-induced expression of Cyclin A2, CDC6, and E2F3a, which are pivotal E2F target gene products involved in cell proliferation (Figure 3D), suggesting that expression of E1a generated deregulated E2F activity in HFFs. Furthermore, the expression of E1a facilitated cell cycle progression in serum-starved HFFs, indicating that E1a induced an oncogenic phenotype in normal HFFs (Figure 3E). To demonstrate that Ad-ERE73s-ARF (−13)-TK can kill E1a-expressing HFFs, we co-infected HFFs with Ad-ERE73s-ARF (−13)-TK along with Ad-12SE1a (Δ2-11). Ad-ERE73 (1 + 2)-ARF (−13)-TK and ERE73 (3 + 4)-ARF (−13)-TK displayed a cytotoxic effect specifically in the presence of E1a expression (Figure 3F). Consistent with this, expression of the *HSV-TK* gene was induced by E1a expression (Figure 3G), likely due to the resulting concomitant deregulated E2F activity. These results provide evidence that ERE73 (1 + 2)-ARF (−13) and ERE73 (3 + 4)-ARF (−13) are promising promoter constructs to selectively drive cytotoxic gene expression in cancer cells, leading to cancer cell-specific killing in vitro.

### 3.4. Ad-ERE73 (1 + 2)-ARF (−13)-TK and ERE73 (3 + 4)-ARF (−13)-TK Show Anti-Tumor Effects In Vivo

**Figure 4:**
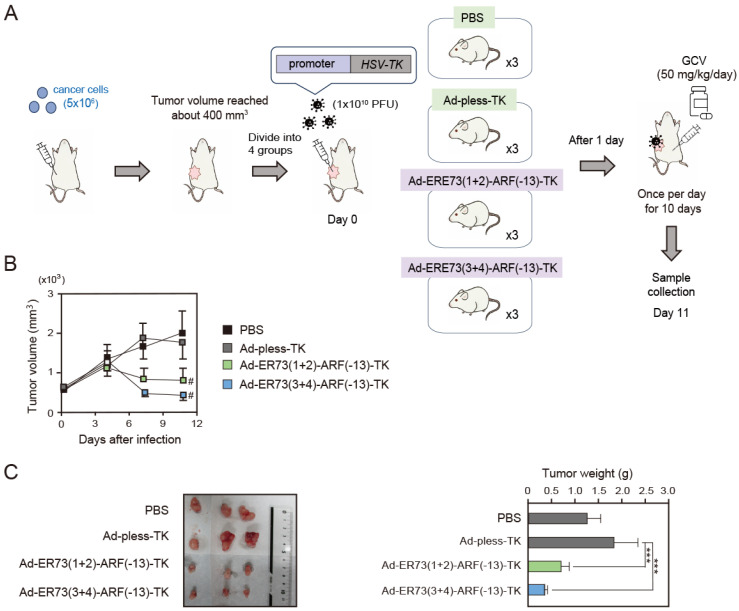
Ad-ERE73 (1 + 2)-ARF (−13)-TK and Ad-ERE73 (3 + 4)-ARF (−13)-TK exhibit anti-tumor effects in vivo. (**A**) Schematic presentation of the experimental protocol and timeline. Cultured DLD-1 cells (5 × 10^6^ cells) were injected subcutaneously into the right flank of athymic 6-week female BALB/c nu/nu mice. When average tumor volume reached about 400 mm^3^, mice were randomly assigned and their tumor was injected with PBS, Ad-pless-TK, Ad-ERE73 (1 + 2)-TK, or Ad-ERE73 (3 + 4)-TK (1 × 10^10^ PFU in 0.1 mL PBS) (3 mice per group). Beginning one day post infection, Ganciclovir (50 mg/kg) was administrated intraperitoneally once per day for 10 days. (**B**) Tumor volumes were monitored for 10 days after adenoviral infection. # *p* < 0.01 compared to Ad-pless-TK. (**C**) Tumor weights were measure at 11 days after infection. *** *p* < 0.01.

To explore the potential application of ERE73s-ARF (−13) constructs in cancer cell-specific gene therapy, we next examined the anti-tumor effects of Ad-ERE73 (1 + 2)-ARF (−13)-TK and Ad-ERE73 (3 + 4)-ARF (−13)-TK in a xenograft mouse model (Figure 4A). Since ERE73s-ARF (−13) constructs showed lower promoter activity than the E2F1 promoter in all cancer cell lines tested (Figure 1E), we wanted to determine whether activity of ERE73s-ARF (−13) constructs is sufficient to drive *HSV-TK* gene expression and suppress tumor growth in vivo. For this purpose, we used DLD-1 cells, in which ERE73s-ARF (−13) showed the lowest cancer cell specificity among the cancer cell lines tested (Figure 2C,D and Figure 4A). DLD-1 cells (5 × 10^6^ cells) were injected subcutaneously into the right flank of athymic 6-week female BALB/c nu/nu mice. The use of athymic 6- to 8-week female BALB/c nu/nu mice is a standard method for mouse xenograft model. This is because they maintain good condition during the experimental period, are less susceptible to hormonal changes, and readily engrafted tumor cells due to their rapid growth. When average tumor volume reached about 400 mm^3^, the tumors were injected with either Ad-pless-TK, Ad-ERE73 (1 + 2)-ARF (−13)-TK, Ad-ERE73 (3 + 4)-ARF (−13)-TK (1 × 10^10^ PFU), or PBS (3 mice per group). Beginning one day after injection, Ganciclovir (50 mg/kg/day) was administered intraperitoneally once per day for 10 days (Figure 4A) and tumor size (volume) was monitored through day 11. Treatment with Ad-ERE73 (1 + 2)-ARF (−13)-TK or Ad-ERE73 (3 + 4)-ARF (−13)-TK significantly reduced tumor volumes as compared to Ad-pless-TK or PBS (Figure 4B). Tumor weight was also significantly reduced by Ad-ERE73 (1 + 2)-ARF (−13)-TK or Ad-ERE73 (3 + 4)-ARF (−13)-TK as compared to Ad-pless-TK or PBS (Figure 4C). These results indicate that the ERE73s-ARF (−13)-TK constructs have the capacity to drive *HSV-TK* gene expression to suppress tumor growth in vivo.

**Figure 5:**
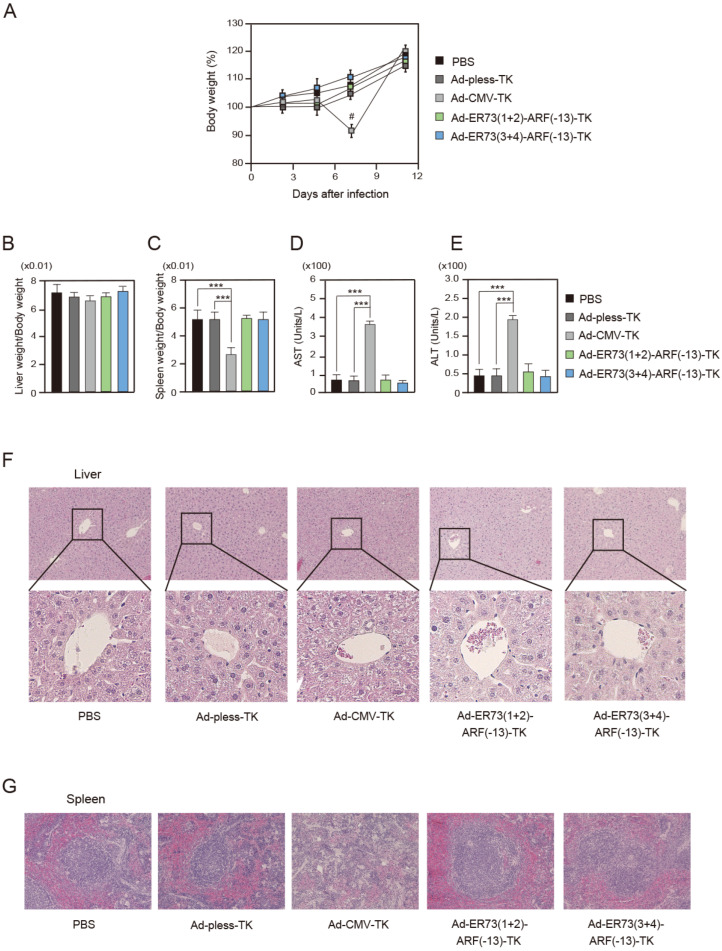
(**A**–**E**) After 11 days of infection of Ad-pless-TK, Ad-CMV-TK, Ad-ERE73 (1 + 2)-TK, or Ad-ERE73 (3 + 4)-TK, body weight (%) (**A**), # *p* < 0.01, liver weight (**B**), spleen weight (**C**), and blood levels of AST (**D**) and ALT (**E**) were examined. *** *p* < 0.01. (**F**,**G**) At day 11, histology of the liver (**F**) and spleen (**G**) was examined after Hematoxylin and Eosin staining.

We also assessed potential side effects of Ad-ERE73 (1 + 2)-ARF (−13)-TK and ERE73 (3 + 4)-ARF (−13)-TK using Ad-CMV-TK, in which the *HSV-TK* gene expression is driven by a strong CMV promoter, as a positive control. The administration of Ad-ERE73 (1 + 2)-ARF (−13)-TK and Ad-ERE73 (3 + 4)-ARF (−13)-TK did not significantly affect body weight (Figure 5A). Although Ad-CMV-TK reduced body weight at day 7, it returned to similar levels to other viral constructs at day 11, suggesting that none of the viruses significantly affected body weight (Figure 5A). Adenoviruses are known to exhibit specific tropism for the liver and secondarily for the kidney in vivo, suggesting that Ad-ERE73 (1 + 2)-ARF (−13)-TK and Ad-ERE73 (3 + 4)-ARF (−13)-TK could have an effect on the liver or the spleen. Neither virus affected liver weight, while Ad-CMV-TK reduced spleen weight, an effect not observed with Ad-ERE73 (1 + 2)-ARF (−13)-TK or Ad-ERE73 (3 + 4)-ARF (−13)-TK (Figure 5B,C). Although Ad-CMV-TK did not reduce liver weight, it increased the blood levels of AST and ALT, which are common markers of hepatic damage (Figure 5D,E). In contrast, Ad-ERE73 (1 + 2)-ARF (−13)-TK, or Ad-ERE73 (3 + 4)-ARF (−13)-TK had no effect on AST and ALT levels (Figure 5D,E). Histopathological analysis revealed that neither virus showed obvious effects on the liver, consistent with no significant effect on liver weight. However, Ad-CMV-TK exhibited a cytopathic effect on the spleen, characterized by loss of follicular structure in white pulp, consistent with reduced spleen weight, whereas Ad-ERE73 (1 + 2)-ARF (−13)-TK and Ad-ERE73 (3 + 4)-ARF (−13)-TK had no such effects (Figure 5F,G). Taken together, these observations indicate that Ad-ERE73 (1 + 2)-ARF (−13)-TK and Ad-ERE73 (3 + 4)-ARF (−13)-TK could effectively reduce tumor cell growth without obvious systemic side effects.

## 4. Discussion

For suicide gene therapy and oncolytic virotherapy, an ideal promoter should have low activity in normal cells to avoid side effects but be highly active in a wide variety of cancer cell types for therapeutic efficacy. In this study, we demonstrated that the cancer cell specificity of ERE73 (1 + 2)-ARF (−13) and ERE73 (3 + 4)-ARF (−13) promoter constructs is significantly greater than that of the native ARF promoter and the commonly used E2F1 and hTERT promoters (Figure 1E). The enhanced cancer specific activity of the ERE73 (1 + 2)-ARF (−13) and ERE73 (3 + 4)-ARF (−13) constructs is mediated by deregulated E2F activity, since it was abolished by mutation of E2F binding sites of ERE73s (Figure 2D), and introduction of a constitutively active form of pRB in cancer cell lines, to suppress E2F, diminished the activities of ERE73s-ARF (−13) promoters (Figure 2E). Recombinant adenovirus expressing *HSV-TK* under the control of ERE73 (1 + 2)-ARF (−13) and ERE73 (3 + 4)-ARF (−13) induced efficient *HSV-TK* gene expression in cancer cell lines but not in normally growing cells (Figure 3B), and showed cancer cell-specific cytotoxicity in vitro and in vivo (Figure 3A and Figure 4B,C). These observations support the notion that the promoter elements of E2F target tumor suppressor genes are useful to drive gene expression specifically in cancer cells.

E2F1 and hTERT promoters have been utilized to regulate gene expression in a cancer cell-specific manner. E2F1 is a target gene of E2F, while hTERT is a target gene of c-Myc. Considering the enhanced expression of E2F and c-Myc in various cancers, the high promoter activity could be mediated by these factors. However, E2F and c-Myc are also expressed in normally growing cells, wherein both their respective promoters can be activated. This observation is consistent with the fact that E2F1 and the hTERT promoters can be activated by growth stimulation in normal cells [21,31], and thus have the potential to impact normally growing cells. In contrast, the ARF and TAp73 promoters are activated by deregulated E2F activity, induced by forced inactivation of pRB, a prototypical oncogenic change, but not by physiological E2F activity, induced by growth stimulation [38,39]. In almost all cancers, the RB pathway is disabled [30], generating deregulated E2F activity, which induces ARF and TAp73 gene expression [38,39,40]. However, the p53 pathway is also disabled [30], and hence deregulated E2F activity is tolerated in cancer cells. Thus, deregulated E2F activity that activates the *ARF* and *TAp73* genes is expected to be present only in cancer cells, since physiological E2F activity induced by growth stimulation does not activate the *ARF* or *TAp73* gene [38,39]. Hence, it is expected that utilizing deregulated E2F activity using ARF and TAp73 promoter elements provides an advantage over enhanced E2F activity in cancer cells and growth-related E2F target promoters such as E2F1, which shows high activity in normally growing cells. Indeed, in HFFs the ARF promoter is far less active than the hTERT and E2F1 promoters and the ARF (−13) core promoter exhibited even lower activity. Moreover, addition of ERE73s to ARF (−13) did not enhance activity at all, whereas addition of E2 enhancer elements containing typical E2F binding sites greatly increased promoter activity. This indicates that, unlike typical E2F binding sequences in normally growing cells, ERE73s do not respond to physiological E2F activity. In contrast, in cancer cell lines, addition of ERE73s to ARF (−13) greatly enhanced promoter activity, possibly due to deregulated E2F activity. These observations indicate that utilizing deregulated E2F activity in cancer cells by tumor suppressor promoter elements has significant advantages over growth-related E2F target promoters, such as E2F1, regulated by enhanced E2F activity, in order to drive therapeutic gene expression specifically in cancer cells.

Our results suggest that the strength or potency of deregulated E2F activity that activates the *ARF* and *TAp73* genes may vary depending on the status of pRB. ERE73s-ARF (−13) showed higher cancer cell specificity in cancer cell lines 5637 and Saos-2 than in DLD-1 and HLF (Figure 1E). This suggests the possibility that there may be differences in the potency or efficacy of deregulated E2F activity between cell lines. Mutation of the *RB1* gene is reported for 5637 [55], and Saos-2 cells express a truncated pRB, incapable of binding to E2F [56]. In contrast, the pRB status of DLD-1 and HLF is unknown, suggesting the presence of functional pRB and possible defects upstream of pRB in these cell lines. Thus, complete loss of pRB function may generate higher deregulated E2F activity compared to partial dysfunction of pRB caused by defects in upstream regulators. Consistent with their relative cancer cell specificity, Ad-ERE73 (1 + 2)-ARF (−13)-TK and ERE73 (3 + 4)-ARF (−13)-TK exhibited correspondingly higher cytotoxicity in pRB mutant cancer cell lines (5637 and Saos-2) compared to putative pRB wild-type cancer cell lines (DLD-1 and HLF) (Figure 3A). These results suggest that deregulated E2F activity-based therapeutic approaches for cancer treatment will be more effective in cancers with mutation or deletion of pRB than in those retaining wild-type pRB expression.

Consistent with high cancer cell-specific activity of ERE73s-ARF (−13), adenovirus expressing *HSV-TK* under the control of the ERE73s-ARF (−13), Ad-ERE73s-ARF-TK, exhibited higher cytotoxicity in four cancer cell lines compared to normally growing fibroblasts. Ad-ERE73s-ARF-TK also showed higher cytotoxicity in normal fibroblasts transduced with adenovirus E1a, which binds and inactivates pRB, than in primary fibroblasts. Although promoter activity and cancer cell specificity of ERE73s-ARF (−13) in DLD-1 cells were less than those observed in 5637 or Soas-2 cells (Figure 2C,D), Ad-ERE73s-ARF-TK was able to inhibit DLD-1 tumor growth in a mouse xenograft model, in the absence of significant side effects, in vivo (Figure 4 and Figure 5). These observations demonstrate the utility of the promoter elements of E2F target tumor suppressor genes in cancer cell-specific cytotoxic gene expression.

Sequences of E2F-responsive elements of the *ARF* and *TAp73* genes are mainly composed of GC repeats and diverge from the typical E2F binding sequence of growth-related genes (TTT^C^/_G_^G^/_C_CGC) [38,39]. These atypical E2F response elements are specifically activated by deregulated E2F activity but not by physiological E2F activity induced by growth stimulation [38,39]. These observations suggest that deregulated E2F activity is distinct from physiological E2F activity and the molecular mechanisms underlying the generation and functional role of deregulated E2F may be different from those of physiological E2F. Accordingly, E2F strictly depends on its heterodimeric partner DP to bind to and activate the typical E2F binding sequences in growth-related genes [57,58]; in contrast, activation of the *ARF* gene by E2F1 does not depend on DP [40]. Understanding the unique molecular and biochemical characteristics of deregulated E2F regulation of transcription will facilitate utilization of deregulated E2F activity in cancer cells, acting on atypical E2F-responsive elements in tumor suppressor E2F target genes, to specifically target cancer cells, while sparing and protecting normally growing cells, thereby maximizing therapeutic efficacy and avoiding potential side effects.

Although utilizing deregulated E2F activity in cancer cells with tumor suppressor E2F target promoter elements is thought to be cancer cell-specific, further studies would be required to improve the system. For example, although cancer cell specificity of the ERE73s-ARF (−13) constructs was higher than that of commonly used E2F1 and hTERT promoters, the promoter activity of the artificial promoters in cancer cell lines was lower than that of the E2F1 promoter, likely due to low basal activity of ARF (−13) core promoter. In addition, the potency or efficacy of deregulated E2F activity in cancer cells may vary depending on the status of pRB and possibly p53. Thus, there might be a limitation in the utility of current ERE73s-ARF (−13) constructs and the activity of artificial promoters in cancer cells needs to be improved. We have identified other tumor suppressor E2F targets, which are specifically activated by deregulated E2F, such as *BIM*, *RASSF1*, *PPP1R13B*, *JMY*, *MOAP1*, *RBM38*, *ABTB1*, *RBBP4*, and *RBBP7* [59]. It would be worth testing whether combination of these tumor suppressor gene promoter elements may create higher promoter activity in cancer cells, while retaining low basal activity in normally growing cells. It is reported that the *ARF* gene is transiently expressed in testes and vitreous during mouse development [60], although it is not known whether this expression is mediated through E2F. Thus, it would also be necessary to examine activity of artificial promoters in other normal cells than HFFs. In this study, we used *HSV-TK* as a suicide gene to test the feasibility of the system. It would be necessary to test the availability of other suicide genes and feasibility in oncolytic gene therapy.

## 5. Conclusions

Utilizing deregulated E2F activity in cancer cells, acting via atypical promoter elements of tumor suppressor E2F target genes, is superior to, and offers significant therapeutic advantages over, the use of enhanced E2F activity, regulating growth-related E2F target gene promoters as a mechanism to drive gene expression specifically in cancer cells.

## Data Availability

The raw data supporting the conclusions of this article will be made available by the authors on request.

## Abbreviations

The following abbreviations are used in this manuscript:

Ad	adenovirus
ALT	alanine transaminase
APC	adenomatous coli
ARF	alternative reading frame
AST	aspartate aminotransferase
Bax	Bcl-2-associated X protein
CDC	cell division cycle
CDK	cyclin-dependent kinase
CMV	cytomegalovirus
DMEM	Dulbecco’s modified Eagle medium
ERE	E2F-responsive element
EREA	E2F-responsive element of ARF promoter
ERE73	E2F-responsive element of TAp73 promoter
FACS	fluorescence activated cell sorting
FCS	fetal calf serum
GAPDH	glyceraldehyde 3-phosphate dehydrogenase
HDM2	human double minute 2
HE	Hematoxylin Eosin
HFF	human foreskin fibroblast
HRP	horse radish peroxidase
HSV	herpes simplex virus
hTERT	human telomerase reverse transcriptase
MOI	multiplicity of infection
MT	mutant type
PEI	Polyethylenimine
PFU	plaque-forming unit
pless	promoter less
RB	retinoblastoma
PBS	phosphate-buffered saline
RL	Renilla luciferase
RPMI	Roswell Park Memorial Institute
RT	reverse transcription
SD	standard deviation
TAp73	transactivating p73
TK	thymidine kinase
WT	wild type
